# Artificial neural networks for HD-sEMG-based hand position estimation: addressing inter- and intra-subject variability

**DOI:** 10.1186/s12984-026-01881-3

**Published:** 2026-02-08

**Authors:** Giovanni Rolandino, Leonardo Lion, Taian Vieira, Ioannis Havoutis, Brian Andrews, James J FitzGerald

**Affiliations:** 1 Nuffield Department of Surgical Sciences, University of Oxford, Oxford, OX3 9DU UK; 2 São Carlos’ School of Engineering and São Carlos’ Institute of Physics, University of São Paulo, São Carlos, 13566–59 SP Brazil; 3 LISiN (Department of Electronics and Telecommunications), Politecnico di Torino, Turin, 10129 Italy; 4 Department of Engineering Science, University of Oxford, Oxford, OX3 9DU UK

**Keywords:** Artificial neural networks, Electromyography, Machine learning

## Abstract

**Background:**

Reliable control of rehabilitation and assistive devices using High-Density surface Electromyography (HD-sEMG) remains limited by poor robustness to electrode shifts, changes in skin condition, and variability across users.

**Methods:**

This study evaluates the performance of the Recursive Prosthetic Control Network (RPC-Net)/High-Density Electrode Array (HDE-Array) system, defined in previous studies, under conditions that reflect real-life usage, including electrode repositioning and cross-subject generalization. The first test evaluated whether the RPC-Net/HDE-Array system maintained stable performance when trained without electrode repositioning and evaluated on data from a different session with altered electrode placement. The study further examined whether explicitly incorporating electrode repositioning during training mitigates the performance degradation typically observed when testing is performed in a separate session. Finally, the effects of inter-subject training were assessed.

**Results:**

Experimental results demonstrate that the RPC-Net/HDE-Array system is highly sensitive to electrode repositioning and skin condition variability when trained under static conditions. However, robustness improves significantly when such variability is included during training. The results indicate that performance improves with an increasing number of subjects in the training pool, provided the training set includes only data from subjects other than the one tested, suggesting a strong dependency on subject-specific patterns

**Conclusions:**

These findings demonstrate that the RPC-Net/HDE-Array system can achieve robust performance across sessions and users when trained under realistic conditions. This work represents a key step toward practical deployment of muscle-computer interfaces.

## Introduction

Despite significant progress in prosthetic design and control strategies, abandonment rates remain high, even for state-of-the-art devices, frequently exceeding 30% [1, 2]. Users cite multiple reasons, including comfort, functional performance, intuitiveness of control, and aesthetics; however, no single factor has been identified as the dominant contributor to abandonment [3–6]. A central challenge is achieving natural control, characterised by isolated finger movements, graded force, effortless daily use, and integrated sensory feedback [7–9]. While hardware has evolved to offer prosthetic hands replicating full human kinematics and Functional Electrical Stimulation (FES) systems targeting individual muscles, many commercial devices still rely on outdated control paradigms, often dating back decades [10–12]. Recent trends and user feedback indicate that improved adoption depends more on advancements in control strategies than on further hardware enhancements [6, 13]. In this context, HD-sEMG has gained attention for its non-invasiveness and the richness of data it provides [14–16].

In previous studies, two components to address current limitations in the control of rehabilitation devices were developed and validated. First, a shallow neural network, Recursive Prosthetic Control Network RPC-Net, that maps High-Density surface Electromyography (HD-sEMG) signals from the proximal forearm to hand kinematics, was introduced [17]. Second, traditional gel electrodes were replaced with the High-Density Electrode Array (HDE-Array) and demonstrated the effectiveness of the HDE-Array in combination with RPC-Net [18]. While preliminary results are encouraging, further validation is essential to confirm real-world applicability.

A critical issue concerns electrode repositioning. In earlier work, as in most similar studies, training and testing datasets were recorded within the same session, typically by splitting a single large dataset. This ensures identical electrode placement during training and testing. However, for practical deployment, users must be able to don and doff the device across sessions, possibly days apart, without sacrificing control accuracy. This implies that the system should maintain robust performance even when trained and tested on data from different sessions, where electrode positions, despite anatomical landmark consistency, may differ. Changes in skin condition likewise affect HD-sEMG signals.

The issues of intra-subject consistency and robustness to changes in electrode position and skin condition have, in recent years, become a central focus within the HD-sEMG community. Several studies quantifying the impact of electrode displacement have reported substantial performance degradation. Du et al. found that convolutional neural network-based classification accuracy dropped by 30–50% when moving from intra-session to inter-session evaluation [19], a result later replicated by Ketykó et al. [20]. Sun et al. further demonstrated that even a 10 mm electrode shift can reduce classification accuracy to 20% [21], with similar magnitudes of decline reported elsewhere [22]. Additional studies consistently confirm that spatial displacement of the electrode array has a major impact on HD-sEMG accuracy [23–25]. While most of these investigations evaluate intra- versus inter-session performance without isolating the contribution of skin-condition changes, some work specifically highlights the detrimental effect of variations at the electrode-skin interface on signal quality and model accuracy [26]. Importantly, nearly all of these studies concern classification rather than regression, where signals are assigned to discrete movement classes rather than mapped continuously to limb kinematics. Only a small number of studies examine inter-session robustness in regression models, and these do so indirectly rather than as a primary objective [27]. Because many of the challenges that affect classification systems also apply to regression, this gap in the literature presents an opportunity for contribution. Collectively, these findings underscore the importance of addressing inter-session consistency, which remains a critical requirement for practical deployment of rehabilitation and assistive systems.

This line of inquiry naturally extends to the broader question of how acquisition-related variability compares with variability arising from inter-subject anatomical differences. Far from being a theoretical consideration, this issue is highly relevant in real-world settings: constructing a multi-subject training dataset that can later be refined for individual users could substantially reduce calibration time and improve usability [28, 29]. Consequently, the effects of inter-subject variability, and approaches for mitigating them, have also received increasing attention in recent years. As with intra-subject variability, much of this work focuses on classification frameworks [30, 31]. Nevertheless, several studies now also investigate inter-subject performance in regression-based decoding, and this research area is growing rapidly [32]. One such study directly quantified inter-subject degradation, reporting a decline from 93.1% intra-subject accuracy to 69.8% when evaluated on unseen users [33]. Among the methods proposed to address both inter-session and inter-subject variability, Transfer Learning (TL) has emerged as a particularly promising approach [34, 35]. TL is a machine-learning framework in which a model trained on a source domain, such as data from other users or sessions, is adapted to a target domain that differs in distribution, enabling the model to maintain high performance with reduced subject-specific training data and improved generalization across users, sessions, or acquisition conditions. This general principle can be implemented in various ways depending on the application domain [34].

Given the importance of robustness to electrode shifts and skin condition changes, as well as the potential of multi-subject training, this work evaluates the RPC-Net/HDE-Array system along these dimensions. Specifically, robustness is assessed by comparing system performance under two conditions: training without repositioning and training with repositioning incorporated. Additionally, the effect of inter-subject training was evaluated by measuring performance when the system is trained on data from multiple users. Four experimental hypotheses regarding the RPC-Net/HDE-Array system were tested:

1. When electrode repositioning is not incorporated in the training session, the accuracy of the RPC-Net/HDE-Array system decreases when the model is tested on data from a different recording session, where variations in electrode placement occur, compared to testing on data from the same session, where electrode positioning remains constant
2. When the RPC-Net/HDE-Array system is tested on data from the same session as the training data, its accuracy remains unchanged regardless of whether electrode repositioning is included during training
3. The performance degradation observed when the system is trained without electrode repositioning and tested on data from a different session can be mitigated by incorporating electrode repositioning during training
4. Including inter-subject data in the training set improves the overall performance of the system, providing a valid alternative to an increased amount of subject-specific data

To test these hypotheses, a new dataset of HD-sEMG and hand-position recordings was acquired, incorporating electrode repositioning during training, and was complemented with previously acquired datasets for comparative analysis. 

## Materials and methods

This section describes the instrumentation and software used in this study (Sect. "Instrumentation") and the experimental procedures employed to assess the hypotheses (Sect. "Experimental Protocol"). All procedures followed the Declaration of Helsinki and received approval from the local ethics committee (CEP Unicamp, Approval Reference: 34583120.2.0000.5404). All data used in this study are available online [36].

### Instrumentation

**Fig. 1:**
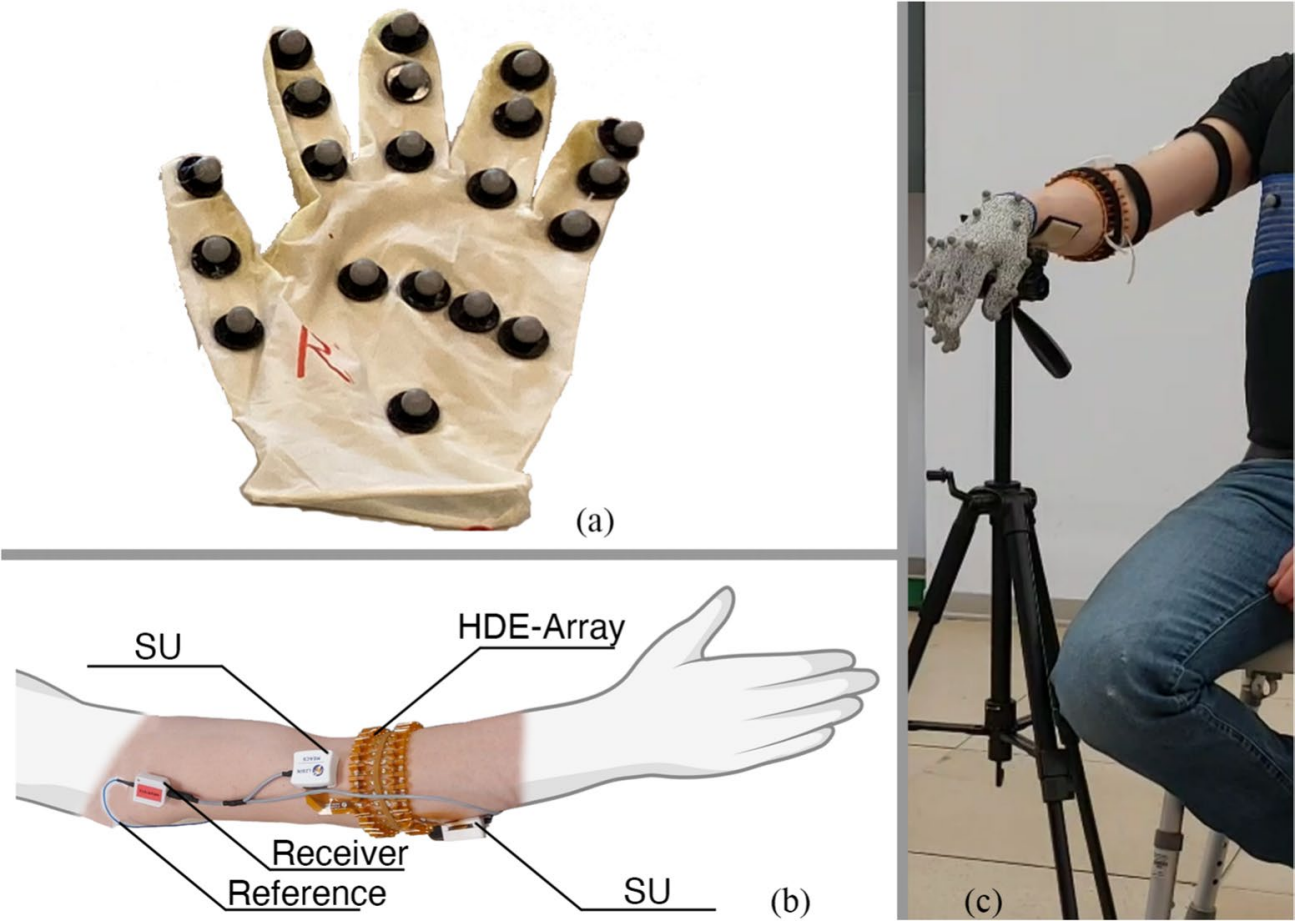
Experimental setup: **a** Motion tracking reflective markers embedded in glove. **b** HDE-Array configuration: The array is shown wrapped around the participant’s forearm. Components are labelled. **c** Full experimental setup is shown. Subject sits wearing the HDE-Array and the glove with embedded markers

Electromyography (EMG) was recorded on the surface of the dominant forearm using the MEACS system, the EMG amplifier developed at LISiN (Politecnico di Torino, Turin, Italy) [37, 38]. The system is made up of multiple Sensor Units (SUs), each measuring 34 mm $\times$ 30 mm $\times$ 15 mm and sampling 32 channels at $f_s$ = 2.048 kHz (192 V/V gain, 16-bit resolution, 2.4 V dynamic range). The modular system can connect to various electrode arrays (e.g. HDE-Array, the array of dry electrodes developed in earlier research and defined in our previous study) [18]. Hand position data were acquired using a motion capture system (Vicon Motus; Vicon Motion Systems, Oxford, UK) sampling at 100 Hz. The setup included 12 infrared cameras (Vero v2.2) and 33 reflective markers. EMG data were acquired using the MEACS system interfaced through BP, a proprietary desktop application. The MEACS system streams raw EMG signals wirelessly to a laboratory desktop computer, where the BP application records, displays and processes them in real time. Motion data were recorded with Vicon Nexus (v2.11, Oxford Metrics plc). To synchronise the EMG and motion tracking data, the MEACS system incorporates a wireless synchronisation unit linked to the Vicon Lock. When motion tracking starts or stops, this unit emits a digital signal recorded alongside the EMG data, facilitating the alignment of the two data sets. A depiction of the instrumentation used for this study, including the HDE-Array and the glove used for motion tracking, is shown in Fig. 1. Subsequent data processing, including filtering and feature extraction, was performed using MATLAB (R2024a, The MathWorks, Inc.), while custom algorithms were implemented in Python, using BSD-licensed libraries.

### Experimental protocol

The objective of the experiments was to assess the hypotheses defined in Sect. "Introduction". The following subsections detail the data acquisition protocol, preprocessing pipeline, network training strategy, testing methodology, and performance evaluation metrics for RPC-Net.

#### Data

Two datasets were used in this study, labelled as DS1 and DS2, which are structurally similar but differ in several key aspects [36]. DS1 was acquired for previous studies, while DS2 was acquired specifically for this study [18, 39]. For clarity and conciseness, the data collection and processing procedures used for DS2 are described and its differences from DS1 are highlighted. For a more thorough description of the procedures involved in the acquisition of DS1, readers are referred to the relevant paper [18]. Table 1 summarises the structure of the two data sets and the difference between them

**Table 1:** Summary of differences between DS1 and DS2.a

	DS1	DS2.a
Subjects	16	4
Date	Nov-Dec 23	Jul-Aug 24
Sessions	2	2
Time between sessions	1–48 hrs	< 30 mins
EMG Channels	64	32
Electrode Repositioning	No	Yes
Skin preparation	Before each session	Between trials 4 and 5

#### DS2.a: joint EMG and kinematic data with electrode repositioning

DS2 comprises two subsets, DS2.a and DS2.b, but only DS2.a was used in the present study. As such, only the procedures relevant to DS2.a are reported. DS2.a comprises data acquired at the Department of Orthopedics, Rheumatology, and Traumatology, State University of Campinas (SP, Brazil), between July and August 2024. Four right-handed healthy volunteers, reporting no surgical interventions on their dominant arm, participated in the study (S29-S32; age: 21 to 28 years; weight: 75 to 90 kg; height: 180 to 195 cm; forearm circumference: 22 to 30 cm). Written informed consent was obtained from all participants. Each subject completed two sessions of simultaneous HD-sEMG and hand kinematic data collection, both performed on the right arm. During the first session (DS2.a.s1), each subject completed six trials; in the second session (DS2.a.s2), they completed two additional trials, for a total of eight trials. During each trial, subjects performed 16 hand poses: 4 wrist poses: flexion, extension, adduction, abduction; 8 finger poses: index finger metacarpophalangeal flexion and extension; index finger proximal interphalangeal flexion and extension; flexion and extension of the middle, ring, and little fingers; adduction and abduction of the index and middle fingers; 4 thumb poses: flexion, extension, adduction, abduction.

**Fig. 2:**
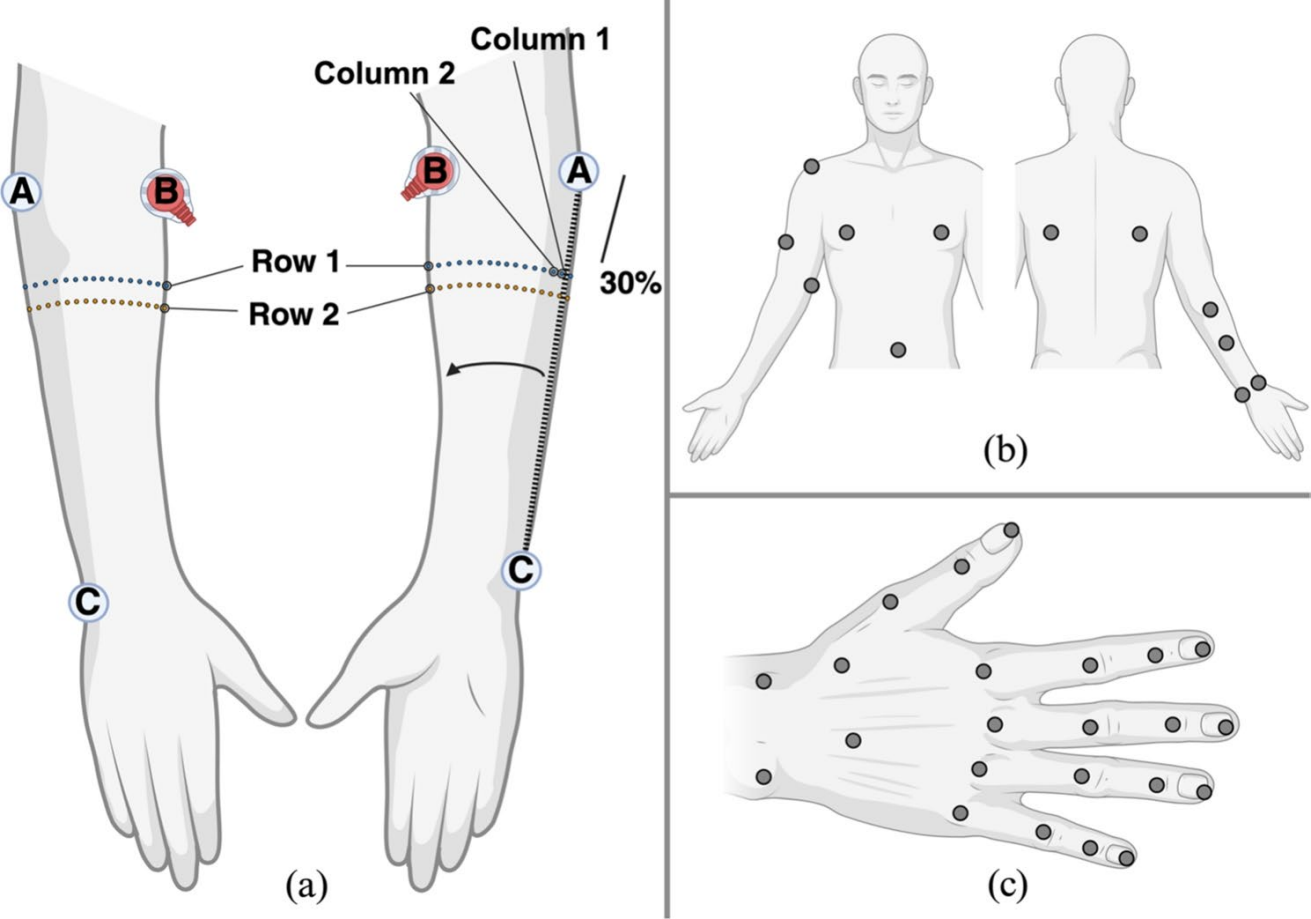
Subject setup: a HDE-Array placement around the forearm, showing the front and back views. Light blue and orange dots represent electrode locations. The array is positioned with electrode 1 at 30% of the distance between the lateral epicondyle and the pisiform bone. b Placement of 12 infrared markers on the subject’s body. c Placement of 21 infrared markers on the hand and 2 on the forearm, with each marker positioned proximally to a joint

**Fig. 3:**
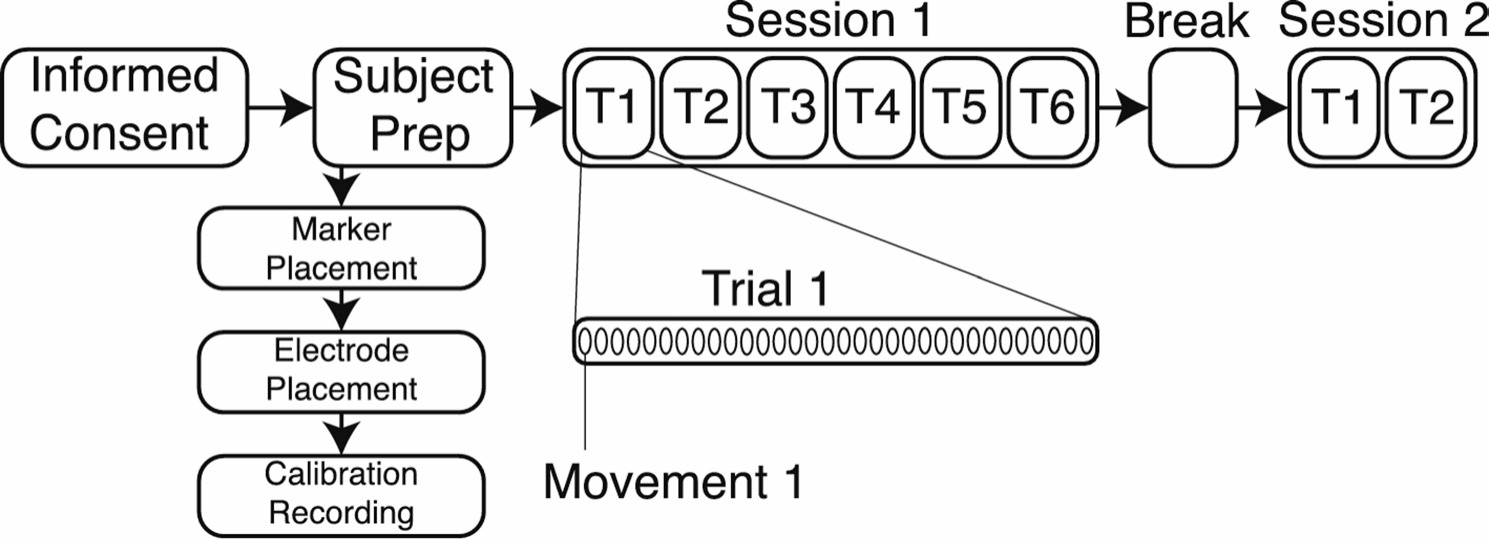
Experimental Protocol Diagram. This figure shows the experimental procedure followed in both DS1 and DS2.a. The preparation steps and the composition of each session is detailed

Before the start of the first session, electrodes and markers were placed. Twenty-one reflective markers were positioned on the dominant hand (embedded in a glove), and 12 more on the upper limb and trunk, for a total of 33 markers (Fig. 2). EMG was acquired using one SU connected to the proximal row of HDE-Array. This resulted in 32 dry electrodes arranged in a single row around the forearm, for a total of *N* = 32 monopolar EMG channels. An additional elastic band around the HDE-Array secured the array and improved skin contact. HDE-Array was positioned so that the proximal electrode of column 1, as defined in Fig. 2, was located at 30% of the distance between the lateral epicondyle and pisiform bone. The reference electrode was placed on the lateral epicondyle. A 10-second recording was performed to calibrate the Vicon system by instructing wrist movements. Each trial consisted of 32 movements (16 poses repeated two times). Participants were seated with their dominant forearm on a vertical support at shoulder height. A monitor prompted poses in random order every 6 to 8 s. The interval between prompts was adjusted by the participants for comfort, without a specified transition speed. Each trial lasted 200 to 260 s, depending on the length of the interval between prompts. To introduce variability in electrode positioning, the HDE-Array was removed and reapplied (following the same anatomical landmarks) between each trial. Additionally, to introduce variability in skin conditions, an abrasive paste was applied between trials 4 and 5 to reduce skin impedance. Trials 1 to 6 constitute the first session. After a break of less than thirty minutes, participants completed two more trials, making up the second session, following the same experimental protocol. Electrodes were again repositioned between trials, though no further skin preparation was performed. These latter two trials constitute DS2.a.s2 as referenced throughout the rest of this manuscript. This process is summarised in Fig. 3.

#### DS1: joint EMG and kinematic data without electrode repositioning

DS1 was acquired as the primary dataset for a previous study [18]. The acquisition procedure is similar to that of DS2.a, with the following key differences: 1) Data were collected at the Laboratory of Motion Analysis (Politecnico di Torino) between November and December 2023. Sixteen healthy right-handed volunteers (S13-S28; 8 males, 8 females; age: 20 to 26 years; weight: 55 to 90 kg; height: 165 to 195 cm) participated. 2) EMG data were acquired using two MEACS SUs connected to both the proximal and distal rows of the HDE-Array, yielding 64 dry electrodes arranged in a single circumferential row around the forearm. When compared with data from DS2.a, only the 32 proximal electrodes are considered. 3) No electrode repositioning was performed during the first session. 4) Skin preparation was carried out before the start of both sessions. 5) The interval between sessions ranged from several hours to two days. 6) In the second session, no electrode repositioning was performed between the two trials. The first six trials, making up session 1, will be referenced as DS1.s1, and the final two (session 2) as DS1.s2.

**Fig. 4:**
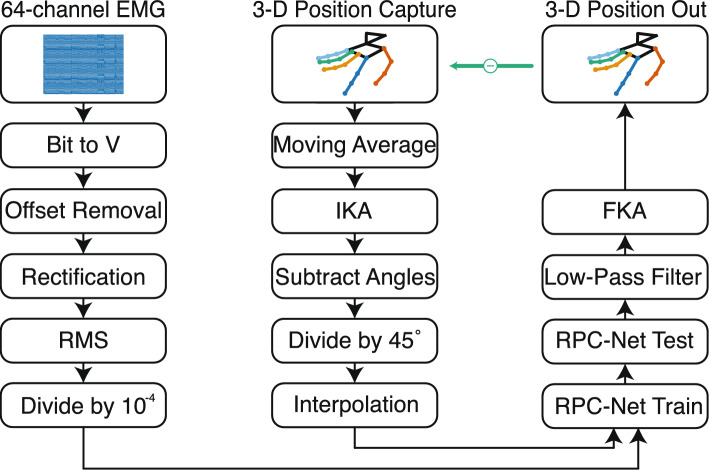
Processing pipeline diagram. This figure shows the procedure followed in this study to pre-process the data, train RPC-Net, and post-process the data. The green arrow in the top-right indicates the comparison made for performance assessment

#### Pre-processing

The pre-processing procedure is intended to transform the acquired data into suitable inputs for RPC-Net. Raw EMG signals were first converted from bits to volts, then offset-corrected, rectified, and transformed into Root Mean Square (RMS) values using a sliding window of $w_l$ = 200 samples (97.7 ms). The window slide $w_s$ was adjusted based on prompt duration, as defined in Sect. "DS2.a: Joint EMG and Kinematic Data with ElectrodeRepositioning": 25 samples for 8 s, 29 for 7 s, and 33 for 6 s, ensuring consistency in sample number across subjects. Given an electromyographic signal that is *L* seconds long and *N*-dimensional, the output of the post-processing procedure is $l = floor(\frac{L*f_s-w_l}{w_s})+1$ samples long and *N*-dimensional. The resulting RMS values were divided by $10^{-4}$ to bring the data into a standardised range suitable for RPC-Net training, resulting in unit variance.

Vicon marker positions were processed using a 20th-order moving average filter and subsequently mapped into a 29-dimensional joint-angle space using the Inverse Kinematic Algorithm (IKA). To prepare the joint-angle data for network training, rest angles were subtracted, and the resulting values were normalised by dividing by 45 degrees, centring the data at zero and resulting in unit variance. Finally, linear interpolation was applied to align the sampling rates of the EMG and joint-angle data, ensuring synchronised, time-matched input–output pairs for RPC-Net training. Given an *L*-second long signal, the output of the post-processing procedure is $l = floor(\frac{L*f_s-w_l}{w_s})+1$ samples long and *J*-dimensional. Pre-processing was identical for DS1 and DS2.a. The pre-processing procedure is depicted in Fig. 4.

#### Kinematic model

RPC-Net requires joint angles as input and returns those in output, so the IKA was developed in a previous study to translate 3D marker positions, as captured by the motion capture system, into 29 hand joint angles [18]. The core of the IKA is an optimisation process designed to identify the 29 joint angles (one per kinematic Degree of Freedom (DoF)) that best approximate the marker positions, executed for each frame captured by the Vicon system. The IKA projects from a 3D space to a *J*-dimensional joint angle space (*J* = 29, number of joints). A Forward Kinematic Algorithm (FKA) was developed to convert joint angles into marker positions.

#### RPC-net training and post-processing

RPC-Net, as defined in our previous study, is a neural network that estimates hand position from HD-sEMG signals acquired on the proximal forearm, refining estimates recursively based on prior values [17]. The version of RPC-Net used in this study processes inputs from 64 (or 32) EMG channels and 29 joint angles. Each sub-network, responsible for a specific joint angle, excludes its corresponding input channel, using the remaining 28 angles instead. This setup creates a robust recursive loop between EMG signals and past angles. During training, actual joint angles replace past estimates. During the testing phase, joint angles produced by the RPC-Net undergo processing via a fourth-order low-pass Butterworth filter (with a cutoff frequency $f_c$ = 1 Hz) to eliminate high-frequency fluctuations and are subsequently mapped back into 3D space using the FKA.

In this study, two versions of RPC-Net were used: RPC-Net-32, which uses 32 EMG channels as input, and RPC-Net-64, which processes 64 channels. RPC-Net-32 is used in the part of the study evaluating the system’s robustness to electrode displacement and skin condition variability, while RPC-Net-64 is used in the second part, which assesses the impact of multi-subject training. Both versions were implemented in PyTorch. On an Intel(R) Xeon(R) Platinum 8268 Central Processing Unit (CPU) at 2.90 GHz, RPC-Net-32 achieves an average Inference Time (IT) of 1.3 ms (standard deviation: 0.2 ms) across $10^{5}$ iterations. The network was trained with the Adam optimiser (in its PyTorch implementation), learning rate = $10^{-5}$; $\varepsilon$ = $10^{-12}$; $\beta _1$ = 0.9; $\beta _2$ = 0.99; batch size = 2000; loss criterion = MSELoss. The model was trained for 200 epochs. During the training of RPC-Net, the joint angle input data were from the recording, and the recursion loop was not used. RPC-Net-64 achieved an average IT of 4.2 ms (standard deviation: 0.2 ms). Given the larger input size and dataset used for the second part of the study, a batch size of 10,000 was adopted for training.

#### Performance assessment

The performance of RPC-Net was assessed as its ability to estimate the position of the hand from the electromyogram. The performance was measured with two indicators: Mean Pearson Correlation Coefficient (MPCC) and Mean Distance (MD), both computed for the test trial only. MPCC is the mean of the individual Pearson Correlation Coefficient (PCC) obtained from comparing the actual and predicted joint angle values for each of the 29 Degrees of Freedom (DoFs) considered, over the whole test trial. MD is the mean, over the whole test trial, of the distances between index, middle, and thumb tips and their estimates. A thorough definition of MD and MPCC is included in our previous study [17].

#### Assessment of robustness to electrode shifts and skin conditions

To evaluate Hypotheses 1 to 3, the performance of the RPC-Net-32/HDE-Array system under different training and testing conditions was compared. Specifically, it was varied whether electrode repositioning was introduced during training, and whether the system was tested on data from the same session or from a separate session.

Two main assessment configurations are defined: 1) Intra-session assessment: RPC-Net-32 was trained on 5 out of the 6 trials (a 5:1 train-test split) within the same session, namely DS2.a.s1 or DS1.s1, and tested on the remaining trial from that session. 2) Extra-session assessment: RPC-Net-32 was trained on the same 5 trials from DS2.a.s1 or DS1.s1 but tested on one of the two trials from DS2.a.s2 or DS1.s2, respectively. In this case, the same subset of 5 trials used for the Intra-session assessment was used, without adding the sixth one to the training data.

Because DS2.a included electrode repositioning and DS1 did not, these two assessment types yield four distinct cases, defined with a two-letter abbreviation:

1. Intra-session with repositioning (DS2.a): IR.
2. Extra-session with repositioning (DS2.a): ER.
3. Intra-session without repositioning (DS1): IN.
4. Extra-session without repositioning (DS1): EN.

Comparing system performance across these four cases addresses Hypotheses 1 to 3. Additionally, to explore how the amount of training data affects performance (Hypothesis 2), intra-session performance of the system (with and without repositioning) was assessed using subsets of 1, 2, 3, 4, and all 5 training trials, while holding the same test trial fixed. An average was then performed across all possible trial combinations to minimise selection bias.

#### Assessment of generalised training via inter-subject data

This experiment was conducted exclusively using DS1. Since no direct comparison with DS2.a was required, all 64 HD-sEMG channels recorded from the forearm were used. Only intra-session data were considered (6 trials per subject). The goal was to evaluate the generalization capability of RPC-Net-64 when trained on data from multiple subjects. The dataset was split into subsets of 2, 4, 8, or 16 subjects. For group sizes of 2, 4, and 8, subjects were randomly selected into non-exhaustive groupings. Specifically, 8 random groups of 2 subjects, 4 groups of 4 subjects, and 2 groups of 8 subjects were evaluated.

Four training combinations (0, 1, 3, and 5) were considered. For combinations 1, 3, and 5 the training protocol consisted of using all data from subjects in a subgroup (training subjects) except one (testing subject) to train RPC-Net-64. Additionally, a number of training trials (corresponding to the combination identifier) from the testing subject were also included during training. These never included the test trial, which remained the same across all combinations, and was used in the testing phase to assess performance. Regardless of group size, simulations were run such that each subject served as the test subject once per group size. This meant that, for each group size considered, 16 versions of RPC-Net-64 were trained. For each combination, 64 versions of RPC-Net-64 were trained (four group sizes $\times$ 16 subjects). Results were averaged across group sizes (16 data points per size). To contextualize performance, a within-subject baseline in which RPC-Net was trained exclusively on data from the test subject using the same number of trials (i.e., 1, 3, or 5, depending on the combination) was also evaluated. The objective of this assessment is to evaluate if data from other subjects can effectively substitute, during training, data from the testing subject. The aim was to evaluate if, for example, the performance observed when training the system on 5 trials from the testing subject can be replicated by training on just one trial, and supplementing with data from different subjects.

For combination 0, no data from the testing subject were included in training. This combination represents a boundary case, and aims to determine how well the system can perform when trained on data from other subjects only. As per combinations 1,3 and 5, regardless of group size, simulations were run such that each subject served as the test subject once per group size. This meant that, for each group size considered, 16 versions of RPC-Net-64 were trained. Results were averaged across group sizes (16 data points per size).

#### Hypothesis testing

Four hypotheses to evaluate the performance of the RPC-Net/HDE-Array system under varying experimental conditions were tested:

1. When electrode repositioning is not incorporated in the training session, the accuracy of the RPC-Net/HDE-Array system decreases when the model is tested on data from a different recording session, where variations in electrode placement occur, compared to testing on data from the same session, where electrode positioning remains constant. To test this hypothesis, system performance in the IN and EN conditions, as defined in Sect. "Assessment of Robustness to Electrode Shifts and SkinConditions" was compared. A one-sided paired t-test was used to assess whether the mean performance across subjects differed significantly between conditions, using both MD and MPCC as evaluation metrics. The null hypothesis assumed equality between conditions, while the alternative hypothesis reflected the expectation that intra-session performance would be superior. Specifically, the hypotheses tested were $H_0: \mu _{IN} \ge \mu _{EN}$ vs. $H_1: \mu _{IN} < \mu _{EN}$ for MD, and $H_0: \mu _{IN} \le \mu _{EN}$ vs. $H_1: \mu _{IN} > \mu _{EN}$ for MPCC. This reflects the fact that lower MD and higher MPCC indicate better performance. Normality of the distributions was verified with a Shapiro-Wilk test.
2. When the RPC-Net/HDE-Array system is tested on data from the same session as the training data, its accuracy remains unchanged regardless of whether electrode repositioning is included during training. To test this hypothesis, the performance of the system under IN and IR conditions was analysed, across increasing numbers of training trials. For each condition, performance curves were constructed by fitting a non-linear regression model of the form $y = a + b \cdot e^{(cx)}$, where *x* is the number of training trials and *y* is the corresponding performance metric (MD and MPCC). Each curve was fit using 80 data points (16 subjects $\times$ 5 training trial counts). Parameter estimates are reported, and the coefficient of determination ($R^{2}$) was used to assess goodness of fit. To assess whether the IN performance curve generalised to the IR condition, the fitted model was applied to the repositioning data and compared the distributions of residuals using Levene’s test. A non-significant result was interpreted as evidence that the variability in model fit was consistent across conditions, supporting generalizability. As a formal test of performance comparability at full training exposure, a one-sided unpaired t-test on the 5-trial condition was conducted. The alternative hypothesis assumed that performance without repositioning would be superior. Specifically, hypotheses tested were: $H_{0}: \mu _{IN} \ge \mu _{IR}$ and $H_{1}: \mu _{IN} < \mu _{IR}$ for MD; $H_{0}: \mu _{IN} \le \mu _{IR}$ and $H_{1}: \mu _{IN} > \mu _{IR}$ for MPCC. Normality was verified with a Shapiro-Wilk test.
3. The performance degradation observed when the system is trained without electrode repositioning and tested on data from a different session can be mitigated by incorporating electrode repositioning during training. To evaluate this hypothesis, a one-sided paired t-test comparing IR and ER performance was performed. The alternative hypothesis was that IR performance would be superior, reflecting a degradation in performance when transitioning across sessions. Specifically, the hypotheses tested were: $H_{0}: \mu _{IR} \ge \mu _{ER}$ and $H_{1}: \mu _{IR} < \mu _{ER}$ for MD; $H_{0}: \mu _{IR} \le \mu _{ER}$ and $H_{1}: \mu _{IR} > \mu _{ER}$ for MPCC. To assess whether repositioning-aware training mitigates this degradation, the results of this test were compared to the corresponding no-repositioning case. Additionally, a one-sided unpaired t-test to directly compare ER and EN was performed. The alternative hypothesis was that training with repositioning would result in better generalization across sessions. Specifically, the hypotheses tested were: $H_{0}: \mu _{ER} \ge \mu _{EN}$ and $H_{1}: \mu _{ER} < \mu _{EN}$ for MD; $H_{0}: \mu _{ER} \le \mu _{EN}$ and $H_{1}: \mu _{ER} > \mu _{EN}$ for MPCC. Together, these analyses test whether training with electrode repositioning improves the system’s robustness to session-to-session variability. Normality of the distributions was verified with a Shapiro-Wilk test.
4. Including inter-subject data in the training set improves the overall performance of the system, providing a valid alternative to an increased amount of subject-specific data. To evaluate this hypothesis, Spearman’s rank correlation test was performed to assess whether system performance exhibited a monotonic trend with respect to the number of training subjects. This analysis was conducted separately for each of the four experimental combinations (cases 0, 1, 3, and 5), defined by the number of additional training trials from the test subject. For all cases, the hypothesis tested was $H_{0}: \rho = 0$ and $H_{1}: \rho \ne 0$. The result was then interpreted to assess whether the monotonic trend was increasing or decreasing. To support the interpretation of monotonic trends, linear regression models of the form $y = a x + b$ were also fit for each case, where *x* represents the number of training subjects and *y* the performance metric. Finally, to provide a direct comparison of the effects of generalised training, the results observed for combination 5 with 15 additional subjects and with no additional subjects were compared. A two-sided paired t-test: $H_{0}: \mu _{15} = \mu _{1}$ and $H_{1}: \mu _{15} \ne \mu _{1}$ was used for both MD and MPCC. Normality of the distributions was verified with a Shapiro-Wilk test.

## Results

This section presents the results of the experimental evaluations outlined in Sect. "Hypothesis Testing". Specifically, it examines the impact of electrode repositioning and skin-condition variability on system performance, as well as the effectiveness of incorporating such variability during training. Additionally, the section assesses the influence of inter-subject training on the generalization capabilities of the RPC-Net/HDE-Array system.

### Robustness of RPC-net against electrode repositioning

**Fig. 5:**
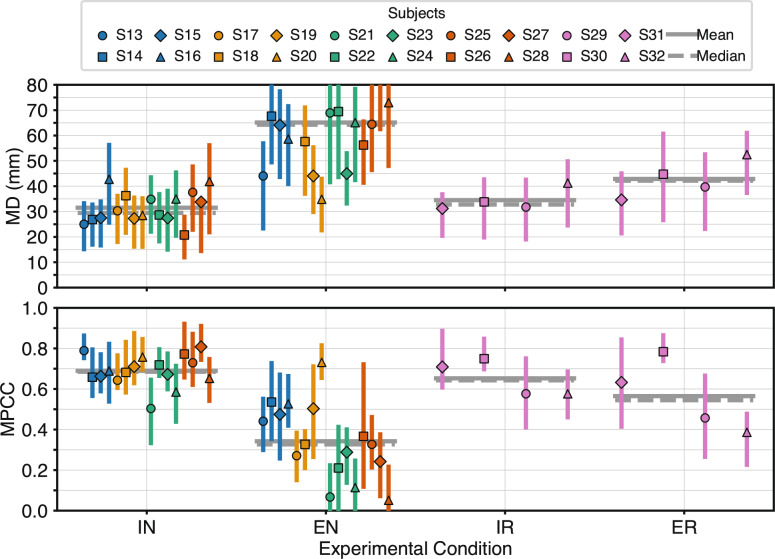
Comparison of RPC-Net performance under different conditions: The figure shows the performance of the RPC-Net/HDE-Array system with and without electrode repositioning and across intra- and extra-session scenarios for all subjects. Interquartile ranges, means, and medians are shown. t-test results for MD: IN vs. EN: $H_{0}: \mu _{IN} \ge \mu _{EN}$; $H_{1}: \mu _{IN} < \mu _{EN}$: *t*(15) = −6.62, *p* = 4.1$\cdot 10^{-6}$; IN vs. IR: $H_{0}: \mu _{IN} \ge \mu _{IR}$; $H_{1}: \mu _{IN} < \mu _{IR}$: *t*(18) = −0.90, *p* = 1.9$\cdot 10^{-1}$; IR vs. ER: $H_{0}: \mu _{IR} \ge \mu _{ER}$; $H_{1}: \mu _{IR} < \mu _{ER}$: *t*(3) = −4.61, *p* = 9.6$\cdot 10^{-3}$; ER vs. EN: $H_{0}: \mu _{ER} \ge \mu _{EN}$; $H_{1}: \mu _{ER} < \mu _{EN}$: *t*(18) = −1.89, *p* = 3.8$\cdot 10^{-2}$. t-test results for MPCC: IN vs. EN: $H_{0}: \mu _{IN} \le \mu _{EN}$; $H_{1}: \mu _{IN} > \mu _{EN}$: *t*(15) = 7.21, *p* = 1.5$\cdot 10^{-6}$; IN vs. IR: $H_{0}: \mu _{IN} \le \mu _{IR}$; $H_{1}: \mu _{IN} > \mu _{IR}$: *t*(18) = 0.83, *p* = 2.1$\cdot 10^{-1}$; IR vs. ER: $H_{0}: \mu _{IR} \le \mu _{ER}$; $H_{1}: \mu _{IR} > \mu _{ER}$: *t*(3) = 1.86, *p* = 8.0$\cdot 10^{-2}$; ER vs. EN: $H_{0}: \mu _{ER} \le \mu _{EN}$; $H_{1}: \mu _{ER} > \mu _{EN}$: *t*(18) = 2.15, *p* = 2.3$\cdot 10^{-2}$

To evaluate whether the performance of RPC-Net decreases when electrode repositioning is included in the testing phase, as posited in Hypothesis 1, EN and IN results were compared. Figure 5 displays performance for four experimental configurations: intra-session and extra-session testing, both with and without electrode repositioning during training. The comparison between intra- and extra-session results without repositioning reveal a substantial deterioration in performance. The MD increased from 31.5 mm to 65.1 mm, while the MPCC dropped from 0.69 to 0.34. Both differences were statistically significant, as confirmed by one-tailed paired t-tests (MD: H1: $\mu _{IN} \le \mu _{EN}$, p = 4.1$\cdot 10^{-6}$; MPCC: H1: $\mu _{IN} \ge \mu _{EN}$, *p* = 1.5$\cdot 10^{-6}$). As a result, Hypothesis 1 is supported: RPC-Net does not maintain consistent performance across sessions in the absence of repositioning-aware training. Summary values for all conditions are provided in Table 2.

**Table 2:** Effect of Electrode Repositioning and Session Variability on RPC-Net Performance

Condition	MD (mm)	MPCC
Repositioning, Intra-Session	34.5 mm	0.65
No Repositioning, Intra-Session	31.5 mm	0.69
Repositioning, Extra-Session	42.9 mm	0.57
No Repositioning, Extra-Session	65.1 mm	0.34

*MD* Mean Distance.* MPCC* Mean Pearson Correlation Coefficient. Lower MD and higher MPCC indicate better performance

### Effect of repositioning on intra-session learning

**Fig. 6:**
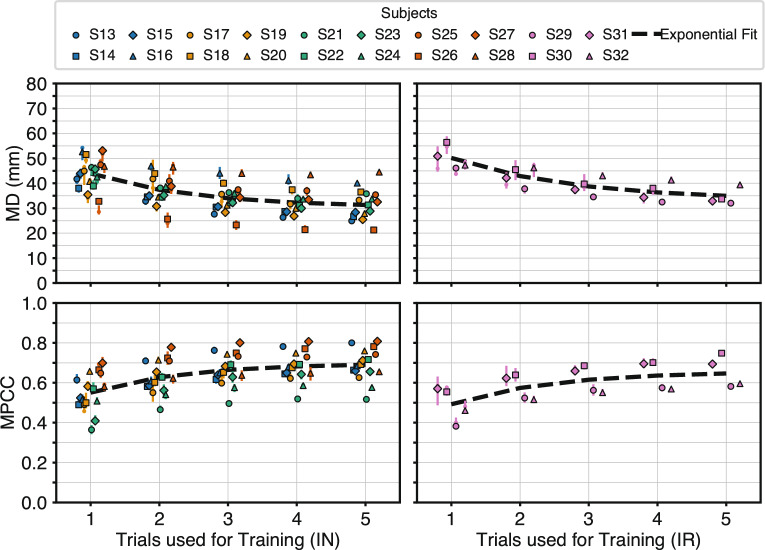
"Intra-session performance with and without electrode repositioning: System performance (MD and MPCC) across all subjects as a function of the number of training trials. Interquartile ranges are shown. Dashed lines represent the best-fit exponential curves ($y = a + b \cdot e^{(cx)}$); estimated coefficients are reported in Table 3. $R^2$ For MD-IN: $R^{2}$ = 0.27; MD-IR: $R^{2}$ = 0.44. For MPCC-IN: $R^2$ = 0.21; MPCC-IR: $R^{2}$ = 0.29. Spearman’s rank test results: MD-IN: $\rho$ = −0.50, *p* = 7.5$\cdot 10^{-33}$; MD-IR: $\rho$ = −0.65, *p* = 4.5$\cdot 10^{-16}$; MPCC-IN: $\rho$ = −0.41, *p* = 6.9$\cdot 10^{-22}$; MPCC-IR: $\rho$ = 0.49, *p* = 6.5$\cdot 10^{-9}$. Mean differences between mean values and exponential estimate: MD-IN: 0.1 mm; MD-IR: 0.2 mm; MPCC-IN: 0.001; MPCC-IR: 0.002. Levene’s test results: For MD, Levene’s statistic = 3.31, *p* = 6.9$\cdot 10^{-2}$; for MPCC, statistic = 0.30, *p* = 5.8$\cdot 10^{-1}$. "

Including electrode repositioning during training was evaluated for its influence on learning dynamics of RPC-Net under intra-session conditions. Specifically, it was assessed whether performance trends as a function of training set size differ in IR and IN. Figure 6 illustrates system performance across increasing numbers of training trials, reported in terms of MD and MPCC. In both the no-repositioning and repositioning conditions, performance improves consistently with additional training data. A relatively stable performance gap is observed across all training set sizes; approximately 5 mm in MD and 0.05 in MPCC, with the no-repositioning condition achieving slightly better absolute values. However, both trends appear to follow similar trajectories in shape. To quantify these trends, the performance data were fit with a non-linear exponential model of the form $y = a + b \cdot e^{(cx)}$, where *x* denotes the number of training trials and *y* the performance metric. For all conditions and metrics, the $R^{2}$ goodness of fit values were computed (MD, no-repositioning: $R^{2}$ = 0.27; MPCC, no-repositioning: $R^{2}$ = 0.21; MD, repositioning: $R^{2}$ = 0.44; MPCC, repositioning: $R^{2}$ = 0.29) and Spearman’s rank correlation test gave: MD, no-repositioning: *p* = 7.5$\cdot 10^{-33}$; MPCC, no-repositioning: *p* = 6.9$\cdot 10^{-22}$; MD, repositioning: *p* = 4.5$\cdot 10^{-16}$; MPCC, repositioning: *p* = 6.5$\cdot 10^{-9}$. These results confirm the presence of a statistically meaningful learning trend with increasing data. Although the fixed-effects curves explain only a modest proportion of pooled variance ($R^{2}$ = 0.21−0.44), this is expected given the design: values were computed on cross-subject data at only five training-set sizes, where large baseline differences between subjects dominate total variance and cannot be captured by a single population curve. Moreover, residuals are heteroscedastic across trial counts, further reducing $R^{2}$ despite a consistent monotonic trend. Because the analysis aimed to describe overall learning behaviour rather than predict individual performance, the stronger indicator is the rank-based Spearman test, which shows highly significant monotonic improvement with additional trials ($p\ll 0.001$). Thus, low pooled $R^{2}$ is compatible with, and does not contradict, the presence of a clear learning effect.

To assess whether the performance model from the no-repositioning condition could generalise to the repositioning data, the fitted no-repositioning model was applied to the repositioning data and compared the resulting residuals using Levene’s test. This analysis evaluates whether the variability (i.e., spread) of residuals differs significantly across conditions, which would indicate model misfit. Levene’s test revealed no significant differences in residual variance for either performance metric (MD: statistic = 3.31, *p* = 6.9$\cdot 10^{-2}$; MPCC: statistic = 0.30, *p* = 5.8$\cdot 10^{-1}$), suggesting that the underlying model structure is applicable to both conditions. The fitted regression parameters for each curve are reported in Table 3. According to these models, approximately 90% of asymptotic performance is reached between 3 and 5 training trials in both cases. Finally, to directly assess end-point performance, a one-sided unpaired t-test was conducted comparing the five-trial results under both training conditions. No statistically significant differences were observed (MD: *p* = 1.9$\cdot 10^{-1}$; MPCC: *p* = 2.1$\cdot 10^{-1}$), indicating that repositioning during training does not significantly affect final performance when sufficient data is available. These findings support Hypothesis 2: RPC-Net achieves comparable intra-session performance regardless of whether electrode repositioning is included in training, particularly when trained on a sufficient number of trials.

**Table 3:** Fitted parameters of the exponential performance model

	No Repositioning		Repositioning	
	MD (mm)	MPCC	MD (mm)	MPCC
a	30.3 ± 2.9	0.70 ± 0.03	33.1 ± 6.3	0.66 ± 0.07
b	26.2 ± 5.6	−0.31 ± 0.09	29.7 ± 7.1	−0.33 ± 0.14
c	−0.66 ± 0.32	−0.74 ± 0.40	−0.56 ± 0.42	−0.68 ± 0.63

Fitted parameters for the exponential model $y = a + b \cdot e^{(cx)}$, where *x* is the number of training trials and *y* is the performance metric (MD or MPCC). Values are shown for intra-session training under both repositioning and no-repositioning conditions

### Effect of repositioning on cross-session performance

To assess whether incorporating electrode repositioning during training mitigates performance degradation across sessions, as stated in Hypothesis 3, IR and ER results were compared. As shown in Fig. 5, the impact of session changes under this condition is notably reduced compared to the no-repositioning case. Although the MD increased from 34.5 mm to 42.9 mm between intra- and extra-session conditions ($H_1$: $\mu _{IR} \le \mu _{ER}$; *p* = 9.6$\cdot 10^{-3}$), the decline in MPCC from 0.65 to 0.57 was not statistically significant ($H_1$: $\mu _{IR} \ge \mu _{ER}$; *p* = 8.0$\cdot 10^{-2}$). These results suggest that incorporating repositioning during training attenuates the negative impact of session-related variability on system performance. This conclusion is further supported by a direct comparison of ER and EN performance. When repositioning was included, performance improved significantly for both MD (42.9 mm to 65.1 mm; $H_1$: $\mu _{ER} \le \mu _{EN}$; *p* = 3.8$\cdot 10^{-2}$) and MPCC (0.57 to 0.34; $H_1$: $\mu _{ER} \ge \mu _{EN}$; *p* = 2.3$\cdot 10^{-2}$). Overall, these results confirm Hypothesis 3: training with electrode repositioning improves the system’s robustness to cross-session variability, reducing performance deterioration in more realistic usage scenarios.

**Table 4:** Fitted parameters for the linear performance model

Case	0	1	3	5
	MD			
a	−1.00	1.77	0.71	0.79
CI (a)	± 0.83	± 0.40	± 0.26	± 0.23
b	80.4	31.0	33.0	28.8
CI (b)	± 7.6	± 3.3	± 2.1	± 1.9
	MPCC			
a	0.005	−0.022	−0.013	−0.013
CI (a)	± 0.004	± 0.005	± 0.005	± 0.004
b	0.20	0.71	0.71	0.75
CI (b)	± 0.03	± 0.04	± 0.04	± 0.03

Parameters and CIs of the equation $ax + b$ for different experimental conditions.* MD* Mean Distance;* MPCC* Mean Pearson Correlation Coefficient

### Effect of inter-subject training on system generalisation

**Fig. 7:**
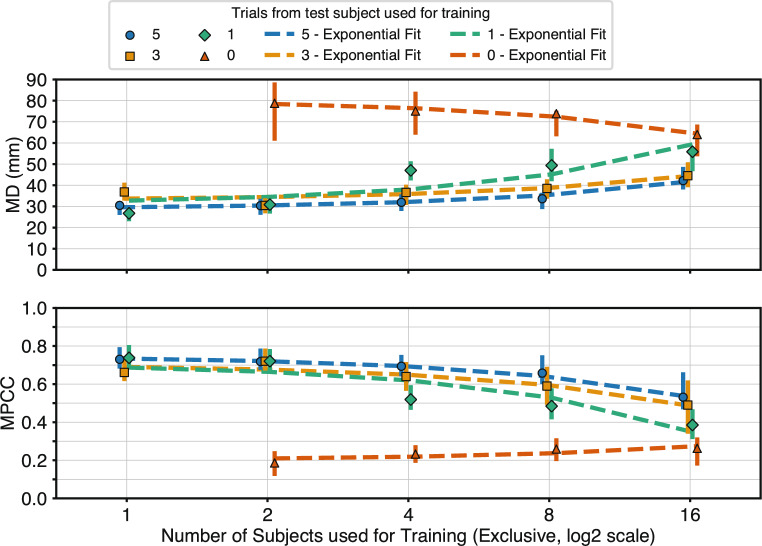
Performance as a function of number of training subjects: Performance trends for Cases 0, 1, 3, and 5 as a function of the number of training subjects. Each marker shows the mean performance across all subjects for the given case; interquartile ranges are indicated. Dashed lines depict the best-fit linear relationships ($y = ax + b$); computed parameters with confidence intervals are in Table 4. The x-axis is plotted on a base-2 logarithmic scale. Regression results: For MD: Case 0: $R^{2}$ = 0.09, *p* = 1.9$\cdot 10^{-2}$; Case 1: $R^{2}$ = 0.50, *p* = 2.6$\cdot 10^{-13}$; Case 3: $R^{2}$ = 0.27, *p* = 8.5$\cdot 10^{-7}$; Case 5: $R^{2}$ = 0.38, *p* = 1.4$\cdot 10^{-9}$. For MPCC: Case 0: $R^{2}$ = 0.10, *p* = 1.3$\cdot 10^{-2}$; Case 1: $R^{2}$ = 0.56, *p* = 1.9$\cdot 10^{-15}$; Case 3: $R^{2}$ = 0.31, *p* = 9.7$\cdot 10^{-8}$; Case 5: $R^{2}$ = 0.36, *p* = 3.4$\cdot 10^{-9}$. Spearman’s rank test results: for MD: Case 0: $\rho$ = −0.30, *p* = 1.8$\cdot 10^{-2}$; Case 1: $\rho$ = 0.83, *p* = 1.1$\cdot 10^{-21}$; Case 3: $\rho$ = 0.41, *p* = 1.4$\cdot 10^{-4}$; Case 5: $\rho$ = 0.51, *p* = 1.6$\cdot 10^{-6}$. for MPCC: Case 0: $\rho$ = 0.35, *p* = 4.5$\cdot 10^{-3}$; Case 1: $\rho$ = −0.81, *p* = 4.9$\cdot 10^{-20}$; Case 3: $\rho$ = −0.47, *p* = 1.3$\cdot 10^{-5}$; Case 5: $\rho$ = −0.52, *p* = 7.3$\cdot 10^{-7}$

**Fig. 8:**
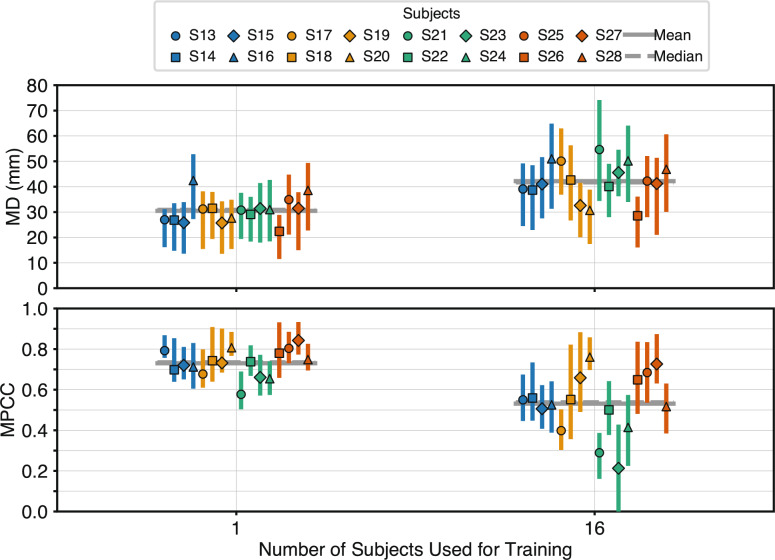
RPC-Net performance with one and sixteen training subjects: The figure shows the performance of the RPC-Net/HDE-Array system in Case 5 when trained with either 15 additional subjects or no additional subjects, across all test subjects considered in this study. The interquartile range is shown, along with the mean and median of each distribution. t-test results for MD: 1 vs. 16: $H_{0}: \mu _{1} = \mu _{16}$; $H_{1}: \mu _{1} \ne \mu _{16}$: *t*(15) = −8.57, *p* = 3.7$\cdot 10^{-7}$. t-test results for MPCC: 1 vs. 16: $H_{0}: \mu _{1} = \mu _{16}$; $H_{1}: \mu _{1} \ne \mu _{16}$: *t*(15) = 8.15, *p* = 6.9$\cdot 10^{-7}$

To assess whether including inter-subject data in the training set improves the overall performance of the system, as stated in Hypothesis 4, performance was evaluated across different training combinations that included varying numbers of subjects. The analysis considered four experimental combinations (Cases 0, 1, 3, and 5), defined by the number of trials from the test subject incorporated into the training set: Case 0: No data from the test subject included; Case 1: One trial included; Case 3: Three trials included; Case 5: Five trials included. Performance trends for each case are shown in Fig. 7. Linear regression models of the form $y = a + bx$ were fitted to describe the relationship between the number of training subjects and system performance, measured by MD and MPCC. The fitted parameters are reported in Table 4. The linear regression revealed a significant positive or negative relationship ($p \le$ 0.05) for all cases. The results reveal that in cases 1, 3, and 5, performance tends to decline as more subjects are added. These trends are confirmed by the results of Spearman’s rank correlation test. For MD, the *p*-values associated with the test $H_0: \rho = 0$ were: 1.1$\cdot 10^{-21}$ (Case 1), 1.4$\cdot 10^{-4}$ (Case 3), and 1.6$\cdot 10^{-6}$ (Case 5). For MPCC, the corresponding values were 4.9$\cdot 10^{-20}$, 1.3$\cdot 10^{-5}$, and 7.3$\cdot 10^{-7}$, respectively. The values of $\rho$ were positive for MD (cases 1,3 and 5), and negative for MPCC. A paired two-sided t-test comparing performance with one subject versus 16 subjects (with five test-subject trials included in both cases) revealed a statistically significant drop in accuracy when adding 15 more subjects (MD: *t*(15) = −8.57, *p* = 3.7$\cdot 10^{-7}$; MPCC: *t*(15) = 8.15, *p* = 6.9$\cdot 10^{-7}$), as illustrated in Fig. 8. These results do not support hypothesis 4, as performance does not improve when including data from a larger pool of subjects. For the sake of completeness, the results derived from Case 0, where no data from the testing subject were included in training, are also reported. The results reveal a divergence in behaviour between Case 0 and the other three cases, as in the former performance improves with the number of additional subjects, reflecting a positive generalization effect. These trends are confirmed by the results of Spearman’s rank correlation test. For MD, the *p*-value associated with the test $H_0: \rho = 0$ was: 1.8$\cdot 10^{-2}$, and 4.5$\cdot 10^{-3}$ for MPCC. The value of $\rho$ was negative for MD and positive for MPCC reinforcing the opposing direction of the performance trend when subject-specific data is present. Only Case 0 aligns with Hypothesis 4 by demonstrating improved performance with increasing subject diversity in the absence of any overlap with test-subject data.

## Discussion

The results presented in the previous section indicate that the RPC-Net/HDE-Array system is not inherently robust to electrode shifts and skin condition variability when such factors are absent from the training data. However, introducing this variability during training significantly mitigates performance degradation, enhancing the system’s robustness in more realistic scenarios. In addition, the findings suggest that inter-subject training does not provide an adequate substitute for additional data from the tested subject. This section provides a detailed interpretation of these findings in relation to the study’s original hypotheses and positions them within the context of current research in the field.

The primary objective of this study, articulated through Hypotheses 1 to 3, was to evaluate the robustness of the RPC-Net/HDE-Array system to electrode repositioning and skin condition variability. The findings presented in Sect. "Robustness of RPC-Net Against Electrode Repositioning" clearly show that, under the previously adopted training protocol, where data are acquired within a single session and without repositioning, the system fails to generalise when electrodes are reapplied. In this condition, endpoint errors exceed 65 mm, a substantial increase compared to the typical performance of under 35 mm. Such a level of inaccuracy renders the system unsuitable for reliable control applications. This degradation is also well below the performance reported in previous work and in other relevant studies, with some subjects exhibiting near-zero correlation between estimated and actual joint angles [17, 18, 40]. These results support the first experimental hypothesis.

However, when variability is intentionally introduced during the training phase, specifically through repeated electrode repositioning and skin preparation, the system becomes substantially more robust, as demonstrated in Sect. "Effect of Repositioning on Cross-Session Performance" . In this configuration, the performance drop between intra- and extra-session testing is reduced to less than 8 mm, with extra-session MD values falling below 45 mm. This performance level is comparable to prior results and aligns with the broader state of the art. Moreover, the 22 mm improvement in MD between the extra-session conditions with and without repositioning-aware training underscores the effectiveness of this approach. Importantly, the electrode repositioning during data collection was performed by the subjects themselves, not by the researchers. This procedure eliminates potential bias and demonstrates the system’s potential robustness in real-world use cases, where users must independently don the system across multiple sessions. Given these results, it is possible to state that the third hypothesis was successfully demonstrated. Additional support for this conclusion comes from Sect. "Effect of Repositioning on Intra-Session Learning" , which shows that including repositioning in the training protocol does not significantly impair intra-session performance. The observed difference, approximately 5 mm in MD, and the non-significant *p*-value, suggest that training protocols incorporating variability can still be suitable for both offline validation and real-time control scenarios. These results support the second hypothesis of the present study. Lastly, while the results presented here may appear slightly worse than those previously reported on the same dataset in a previous study, this discrepancy is attributable to a methodological change: in this part of the current study, only a single row of electrodes was used, whereas previous work employed both proximal and distal rows [18]. The findings presented in this part of the study are particularly important for the development of user-friendly, deployable assistive technologies, where users cannot rely on precise electrode reapplication by trained personnel. In real-life scenarios, individuals must be able to maintain performance over time and operate the device as independently as possible. Ensuring robust performance under self-operated conditions significantly lowers the barrier to adoption in both clinical and home settings, making long-term rehabilitation and daily assistive use more feasible. The results demonstrate that the RPC-Net/HDE-Array system can support this level of robustness.

The present findings are consistent with prior reports on strategies to mitigate inter-session variability. For example, Du et al. observed an increase in classification accuracy of 30% with deep domain adaptation across sessions, and Ketykó et al. reported a 77% improvement using a two-stage RNN with a learned linear adaptation layer [19, 20]. Direct comparison with our results, however, is not straightforward for two reasons. First, both studies address classification, so their performance metric (accuracy) is not directly comparable to the distance- and correlation-based metrics used here for continuous kinematic regression. Second, those approaches implement post hoc, algorithmic domain adaptation, whereas our strategy is data-centric: we expand the training distribution by acquiring data across multiple electrode placements and skin conditions so that the model learns the variability directly. Closer to our approach, Kim et al. evaluated how performance changes when training on different numbers of donning-doffing sessions [23]; in this study, moving from multi-session training (4–9 sessions) back to single-session training typically costs 8–15 percentage points of accuracy, depending on the posture set. Furthermore, Pereira et al. showed that training on augmented electrode positions markedly reduces shift-induced degradation, standard training loses 25% accuracy under electrode shift, whereas training on all channel subsets limits the drop to 2–4% and improves session-to-session performance by 4–7% [22]. Taken together, this evidence supports the central conclusion of our work: explicitly representing expected variability, whether via post hoc adaptation or by training on a broadened, multi-domain dataset, substantially improves robustness in HD-sEMG-based control systems.

The second objective of this study, addressed through Hypothesis 4, was to evaluate whether including data from additional subjects during training could improve the performance of the RPC-Net/HDE-Array system. As shown in Sect. "Effect of Inter-Subject Training on System Generalisation", such improvement was observed only when no data from the test subject were included in the training set (Case 0). This finding suggests that the model is highly sensitive to subject-specific patterns in the sEMG data. When personalised data are available, they appear to dominate the learning process; introducing data from other individuals may dilute their influence, thereby reducing overall performance. An exception can be observed in the fact that, when using three trials from the test subject, performance appears to improve when an additional subject is included in the training set. This result, while representing an exception, indicates that, in some cases, the addition of another subject can bring improvements. Conversely, when subject-specific data are unavailable, the addition of inter-subject data likely enables the model to learn more generalizable patterns of activation. In this case, performance improves with the number of training subjects, likely due to increased exposure to variability across individuals. Still, across all conditions tested, the inclusion of even a small amount of data from the target subject consistently yielded better results than purely inter-subject training. Interestingly, the performance trend observed in Fig. 7 appears linear in model space, though the logarithmic scaling of the plot may visually exaggerate early improvements. This suggests the possibility that, with a sufficiently large training pool, inter-subject training alone could eventually outperform combinations that rely on limited subject-specific data. Future work will focus on expanding the number and diversity of subjects to explore this direction.

Our inter-subject findings are consistent with prior work showing that naïve pooling across users rarely substitutes for even limited personalisation. In classification, cross-subject evaluation typically incurs a substantial penalty relative to subject-specific models [30, 31], with recent reports quantifying drops from 93.1% to 69.8% when moving to unseen users [33]. For continuous decoding, cross-subject regression likewise shows reduced correlation and higher error unless some form of user adaptation or transfer is applied [32]. In our data, adding other subjects helped only when no target-subject data were available (Case 0), but degraded performance once even small amounts of target-subject data were present (Cases 1/3/5), mirroring the conclusion that broad pretraining is useful, yet lightweight personalisation remains critical. Together with recent transfer-learning studies [34, 35], these results suggest a hybrid path forward: learn a general prior from diverse users, then retain a brief, low-burden calibration step to capture subject-specific patterns. In summary, the findings suggest that the RPC-Net/HDE-Array system currently relies heavily on subject-specific data and does not reliably benefit from inter-subject learning when such data are available, thus only partially demonstrating the fourth hypothesis of this study. The development of a successful inter-subject training protocol would be of paramount importance for real-time applications because it would, potentially, remove the need for subject-specific training, giving end-users a device ready for use without the need for refinement. Further studies will aim to achieve this result.

## Conclusion

This study validated the performance of the RPC-Net/HDE-Array system under realistic conditions, where assumptions of consistent electrode placement and stable skin conditions no longer hold. The results demonstrate that, while the system is sensitive to such variability when unaccounted for, deliberately introducing it during training significantly enhances robustness. This supports the feasibility of using an initial training phase, based on motor tracking, to enable consistent control in subsequent sessions without retraining. The potential of inter-subject training to improve performance was also evaluated. While promising only in the absence of test-subject data, these results show that RPC-Net can generalise from multi-subject datasets to achieve satisfactory performance. Developing a robust protocol for effective inter-subject training remains an important goal. If achieved, it would reduce time-consuming subject-specific calibration, providing users with a ready-to-use interface for intuitive and natural control.

Several limitations temper the generalisability of our findings. First, we used two datasets with distinct acquisition setups and session structures (Sect. "DS2.a: Joint EMG and Kinematic Data with ElectrodeRepositioning" and Sect. "DS1: Joint EMG and Kinematic Data without ElectrodeRepositioning"). DS2.a (used for repositioning analyses) employed a single circumferential row of 32 dry electrodes with repeated subject-led donning/doffing between trials and a short break between sessions (less than 30 min). It also introduced a controlled change in skin condition (abrasive paste) midway through Session 1. By contrast, DS1 (used for the no-repositioning baseline and for inter-subject analyses) originally recorded 64 channels (two rows), performed skin preparation before both sessions, and featured longer inter-session intervals (hours to two days), with no repositioning during sessions. Although, for fairness, only the 32 proximal channels of DS1 were considered when directly compared to DS2.a in the repositioning analysis, residual differences remain and may have influenced effect sizes. In particular:

- *Inter-session interval* The shorter pause in DS2.a could have reduced physiological drift (e.g., hydration, perspiration, impedance changes) relative to DS1. As a result, the extra-session performance drop observed with repositioning-aware training in DS2.a (typically $<8$ mm MD) may underestimate the degradation expected over multi-day intervals. Conversely, the larger extra-session drop in DS1 (without repositioning) may reflect a combination of time-dependent skin/interface changes and session-to-session variability, not solely a lack of repositioning-aware training.
- *Skin-condition protocol* DS2.a deliberately altered skin impedance once within Session 1, whereas DS1 standardised skin preparation at the start of both sessions. These differences help test robustness under realistic conditions but confound a strict attribution of performance changes to repositioning alone. Disentangling the isolated effects of electrode shift versus skin-interface changes will require a factorial design that manipulates them independently across matched session intervals.
- *Sample size and statistical power* Repositioning-aware cross-session comparisons involved four participants (DS2.a; see IR vs. ER, *t*(3)), limiting the precision of effect-size estimates and increasing the risk of type II error for some outcomes (e.g., MPCC in cross-session tests). By contrast, no-repositioning comparisons (DS1; IN vs. EN) involved 16 participants, yielding higher power but also reflecting a different session structure. Future work will increase the number and diversity of participants under each condition to harmonise power across analyses.

Further constraints include: (i)* Population* all participants were able-bodied and right-handed; generalisation to clinical populations (e.g., individuals with limb loss, neuromuscular conditions, or altered muscle physiology) remains to be established. (ii)* Open-loop evaluation* performance was assessed offline; closed-loop, real-time control with functional tasks is needed to quantify usability, stability, and user adaptation. (iii)* Ground-truth kinematics* joint angles were derived from optical motion capture via an inverse-kinematics model; modelling errors and marker artefacts may add noise to MPCC/MD estimates. (iv)* Scope of variability* repositioning in DS2.a did not explicitly control the magnitude and direction of shifts (e.g., circumferential rotation versus proximal-distal displacement), and only a single-row configuration was tested in the robustness experiments; broader shift taxonomies and multi-row configurations may reveal different sensitivities.

To address these limitations, future studies will: (1) unify acquisition protocols across sites (array configuration, session timing, and skin-prep procedures) to isolate the contributions of electrode shift versus time-dependent skin changes; (2) increase cohort size and diversity, including clinical populations; (3) perform closed-loop evaluations with assistive devices and functional tasks; (4) quantify and control specific shift types (rotational vs. longitudinal, magnitude calibration) and test multi-row arrays within the same protocol; and (5) explore hybrid strategies that combine data-centric variability exposure with light-weight subject adaptation to reduce calibration while preserving performance.

Within these constraints, our results demonstrate that representing expected variability during training substantially improves robustness across sessions, and that limited, targeted subject-specific data remain more valuable than broad inter-subject pools for the current RPC-Net/HDE-Array pipeline. By improving robustness, reducing calibration demands, and identifying concrete next steps toward full generalization, this work advances muscle-computer interfaces toward intuitive, ready-to-use solutions that can meaningfully improve the quality of life for individuals with upper-limb impairments.

## Data Availability

The dataset supporting the conclusions of this article is available in the "RPC-Net Dataset. Simultaneous HD-sEMG Recordings on the Forearm and angles of a 29-DoF Hand Kinematic Model" repository, https://zenodo.org/records/14,246,378

## Abbreviations

FES: Functional electrical stimulation
HD-sEMG: High-density surface electromyography
sEMG: Surface electromyography
EMG: Electromyography
RPC-Net: Recursive prosthetic control network
HDE-Array: High-density electrode array
IKA: Inverse kinematic algorithm
FKA: Forward kinematic algorithm
DoF(s): Degree(s) of freedom
MD: Mean distance
PCC: Pearson correlation coefficient
MPCC: Mean pearson correlation coefficient
IT: Inference time
MSE: Mean squared error
CPU: Central processing unit
SU(s): Sensor Unit(s)
CI: Confidence interval

## References

[1] Salminger S, Stino LHPH. Current rates of prosthetic usage in upper-limb amputees - have innovations had an impact on device acceptance? Disability Rehabilit. 2020. 10.1080/09638288.2020.1866684. 10.1080/09638288.2020.1866684 33377803

[2] Biddiss EA, Chau TT. Upper limb prosthesis use and abandonment: A survey of the last 25 years. Prosthet Orthot Int. 2007;31(3):236–57. 10.1080/03093640600994581. 17979010 10.1080/03093640600994581

[3] Cordella F. Literature review on needs of upper limb prosthesis users. Front Neurosci. 2016. 10.3389/fnins.2016.00209. 27242413 10.3389/fnins.2016.00209 PMC4864250

[4] Resnik LJ, et al. Measuring satisfaction with upper limb prostheses: orthotics and prosthetics user survey revision that includes issues of concern to women. Arch Phys Med Rehabil. 2022;103(12):2316–24. 35705138 10.1016/j.apmr.2022.05.008

[5] Kyberd PJ, Hill W. Survey of upper limb prosthesis users in sweden, the united kingdom and canada. Prosthet Orthot Int. 2011. 10.1177/0309364611409099. 21697204 10.1177/0309364611409099

[6] Einfeldt AK, D.Y.e.a. F. Rebmann. What do users and their aiding professionals want from future devices in upper limb prosthetics? a focus group study. PLoS ONE. 2023. 10.1371/journal.pone.0295516. 38157364 10.1371/journal.pone.0295516 PMC10756510

[7] E. Biddiss, T.C.: Upper-limb prosthetics. critical factors in device abandonment. American Journal of Physical Medicine and Rehabilitation (2007) 10.1097/PHM.0b013e3181587f6c 10.1097/PHM.0b013e3181587f6c 18090439

[8] Kerver N, S.v.T.e.a. C.K. van der Sluis. Towards assessing the preferred usage features of upper limb prostheses: most important items regarding prosthesis use in people with major unilateral upper limb absence-a dutch national survey. Disabil Rehabil. 2021. 10.1080/09638288.2021.1988734. 34813394 10.1080/09638288.2021.1988734

[9] Østlie K, Lesjø IM, Franklin RJ, Garfelt B, Skjeldal OH, Magnus P. Prosthesis use in adult acquired major upper-limb amputees: patterns of wear, prosthetic skills and the actual use of prostheses in activities of daily life. Disabil Rehabil Assist Technol. 2012;7(6):479–93. 22315926 10.3109/17483107.2011.653296

[10] Ahmadizadeh C, et al. Human machine interfaces in upper-limb prosthesis control: A survey of techniques for pre-processing and processing of biosignals. IEEE Sig Proc Mag. 2021;38(4):12–22. 10.1109/MSP.2021.3057042.

[11] Marinelli A, Nicolo Boccardo FT. Active upper limb prostheses: a review on current state and upcoming breakthroughs. Progr Biomed Eng. 2023. 10.1088/2516-1091/acac57. 10.1088/2516-1091/acac57 41074852

[12] Cimolato A, et al. Emg-driven control in lower limb prostheses: A topic-based systematic review. J Neuroeng Rehabil. 2022;19(1):43. 35526003 10.1186/s12984-022-01019-1 PMC9077893

[13] Kyberd PJ. Assessment of functionality of multifunction prosthetic hands. J Prosthet Ortoht. 2017;29(3):103–11. 10.1097/JPO.0000000000000139.

[14] Igual C, Pardo LA, Hahne J, Igual JM. Myoelectric control for upper limb prostheses. Electronics. 2019;8(11):1244.

[15] Mendez V, Iberite F, Shokur S, Micera S. Current solutions and future trends for robotic prosthetic hands. Ann Rev Contr Robot Autonomous Syst. 2021;4(1):595–627. 10.1146/annurev-control-071020-104336.

[16] Félix Chamberland, S.T.e.a. Étienne Buteau: Novel wearable hd-emg sensor with shift-robust gesture recognition using deep learning. IEEE TRANSACTIONS ON BIOMEDICAL CIRCUITS AND SYSTEM (2023) 10.1109/TBCAS.2023.3314053 10.1109/TBCAS.2023.3314053 37695958

[17] Rolandino G, Gagliardi M, Cerone GL, Andrews B, FitzGerald JJ. Developing rpc-net: Leveraging high-density electromyography and machine learning for improved hand position estimation. IEEE Trans Biomed Eng. 2023;71(5):1617–27. 10.1109/TBME.2023.3346192 38133970

[18] Rolandino, G., Zangrandi, C., Vieira, T., Cerone, G.L., Andrews, B., FitzGerald, J.J.: Hde-array: Development and validation of a new dry electrode array design to acquire hd-semg for hand position estimation. IEEE Transactions on Neural Systems and Rehabilitation Engineering (2024) 10.1109/TNSRE.2024.3490796 39495692

[19] Du Y, Jin W, Wei W, Hu Y, Geng W. Surface emg-based inter-session gesture recognition enhanced by deep domain adaptation. Sensors. 2017;17(3):458. 28245586 10.3390/s17030458 PMC5375744

[20] Ketykó, I., Kovács, F., Varga, K.Z.: Domain adaptation for semg-based gesture recognition with recurrent neural networks. In: 2019 International Joint Conference on Neural Networks (IJCNN), pp. 1–7 (2019). IEEE

[21] Sun, T., Libby, J., Rizzo, J., Atashzar, S.F.: Deep augmentation for electrode shift compensation in transient high-density semg: Towards application in neurorobotics. In: 2022 IEEE/RSJ International Conference on Intelligent Robots and Systems (IROS), pp. 6148–6153 (2022). IEEE

[22] Pereira, J., Halatsis, D., Hodossy, B., Farina, D.: Tackling electrode shift in gesture recognition with hd-emg electrode subsets. In: ICASSP 2024-2024 IEEE International Conference on Acoustics, Speech and Signal Processing (ICASSP), pp. 1786–1790 (2024). IEEE

[23] Kim J, et al. Semg-based hand posture recognition considering electrode shift, feature vectors, and posture groups. Sensors. 2021;21(22):7681. 34833756 10.3390/s21227681 PMC8624257

[24] He J, et al. Position identification for robust myoelectric control against electrode shift. IEEE Trans Neural Syst Rehabil Eng. 2020;28(12):3121–8. 33196444 10.1109/TNSRE.2020.3038374

[25] Li Z, et al. Electrode shifts estimation and adaptive correction for improving robustness of semg-based recognition. IEEE J Biomed Health Inform. 2020;25(4):1101–10. 10.1109/JBHI.2020.3012698 32750979

[26] Abdoli-Eramaki M, Damecour C, Christenson J, Stevenson J. The effect of perspiration on the semg amplitude and power spectrum. J Electromyogr Kinesiol. 2012;22(6):908–13. 22613823 10.1016/j.jelekin.2012.04.009

[27] Bao T, Zaidi SAR, Xie S, Yang P, Zhang Z-Q. A cnn-lstm hybrid model for wrist kinematics estimation using surface electromyography. IEEE Trans Instrum Meas. 2020;70:1–9.33776080

[28] Li J, et al. Deep end-to-end transfer learning for robust inter-subject and inter-day hand gesture recognition using surface emg. Biomed Signal Process Control. 2025;100:106892.

[29] Long Y, et al. A transfer learning based cross-subject generic model for continuous estimation of finger joint angles from a new user. IEEE J Biomed Health Inform. 2023;27(4):1914–25. 37018609 10.1109/JBHI.2023.3234989

[30] Islam, M.R., Massicotte, D., Massicotte, P., Zhu, W.-P.: Surface emg-based inter-session/inter-subject gesture recognition by leveraging lightweight all-convnet and transfer learning. IEEE Transactions on Instrumentation and Measurement (2024)

[31] Tam S, Boukadoum M, Campeau-Lecours A, Gosselin B. Intuitive real-time control strategy for high-density myoelectric hand prosthesis using deep and transfer learning. Sci Rep. 2021;11(1):1–14. 34050220 10.1038/s41598-021-90688-4 PMC8163779

[32] Lin C, He Z. A rotary transformer cross-subject model for continuous estimation of finger joints kinematics and a transfer learning approach for new subjects. Front Neurosci. 2024;18:1306050. 38572147 10.3389/fnins.2024.1306050 PMC10987947

[33] Colot, M., Simar, C., Petieau, M., Cebolla Alvarez, A.M., Cheron, G., Bontempi, G.: Emg subspace alignment and visualization for cross-subject hand gesture classification. In: Joint European Conference on Machine Learning and Knowledge Discovery in Databases, pp. 416–423 (2023). Springer

[34] Wu D, Yang J, Sawan M. Transfer learning on electromyography (emg) tasks: Approaches and beyond. IEEE Trans Neural Syst Rehabil Eng. 2023;31:3015–34. 10.1109/TNSRE.2023.3295453. 37450364 10.1109/TNSRE.2023.3295453

[35] Chen X, et al. Hand gesture recognition based on surface electromyography using convolutional neural network with transfer learning method. IEEE J Biomed Health Inform. 2020;25(4):1292–304. 10.1109/JBHI.2020.3009383 32750962

[36] Rolandino, G., et al.: RPC-Net Dataset. Simultaneous HD-sEMG Recordings on the Forearm and angles of a 29-DoF Hand Kinematic Model. available: https://zenodo.org/records/14246378 (2024). 10.5281/zenodo.14246378

[37] Cerone GL, Botter A, Gazzoni M. A modular, smart, and wearable system for high density semg detection. IEEE Trans Biomed Eng. 2019;66(12):3371–80. 30869608 10.1109/TBME.2019.2904398

[38] Cerone GL, Botter TVA. Design and characterization of a textile electrode system for the detection of high-density semg. IEEE Trans Neural Syst Rehabil Eng. 2021;29(12):1110–9. 10.1109/TNSRE.2021.3086860. 34097613 10.1109/TNSRE.2021.3086860

[39] Rolandino, G., Vieira, T., Cerone, G.L., Andrews, B., FitzGerald, J.J.: Performance of a ML-Based 3-DoF Kinematic Model in Estimating Hand Position from High-Density EMG. In: Proceedings of the IFESS Conference, Bath, UK (2024). Presented at the IFESS Conference

[40] Sîmpetru RC, März M, Del Vecchio A. Proportional and simultaneous real-time control of the full human hand from high-density electromyography. IEEE Trans Neural Syst Rehabil Eng. 2023;31:3118–31. 37440382 10.1109/TNSRE.2023.3295060

[41] Richards A. University of Oxford Advanced Research Computing. Zenodo. 2015. 10.5281/zenodo.22558.

